# DNA–protein cross-link repair: what do we know now?

**DOI:** 10.1186/s13578-019-0366-z

**Published:** 2020-01-07

**Authors:** Huimin Zhang, Yun Xiong, Junjie Chen

**Affiliations:** Department of Experimental Radiation Oncology, The University of Texas MD Anderson Cancer Center, Houston, TX USA

**Keywords:** DNA–protein cross-link, SPRTN, NER, HR, TDP1/TDP2

## Abstract

When a protein is covalently and irreversibly bound to DNA (i.e., a DNA–protein cross-link [DPC]), it may obstruct any DNA-based transaction, such as transcription and replication. DPC formation is very common in cells, as it can arise from endogenous factors, such as aldehyde produced during cell metabolism, or exogenous sources like ionizing radiation, ultraviolet light, and chemotherapeutic agents. DPCs are composed of DNA, protein, and their cross-linked bonds, each of which can be targeted by different repair pathways. Many studies have demonstrated that nucleotide excision repair and homologous recombination can act on DNA molecules and execute nuclease-dependent DPC repair. Enzymes that have evolved to deal specifically with DPC, such as tyrosyl-DNA phosphodiesterases 1 and 2, can directly reverse cross-linked bonds and release DPC from DNA. The newly identified proteolysis pathway, which employs the proteases Wss1 and SprT-like domain at the N-terminus (SPRTN), can directly hydrolyze the proteins in DPCs, thus offering a new venue for DPC repair in cells. A deep understanding of the mechanisms of each pathway and the interplay among them may provide new guidance for targeting DPC repair as a therapeutic strategy for cancer. Here, we summarize the progress in DPC repair field and describe how cells may employ these different repair pathways for efficient repair of DPCs.

## Background

DNA in eukaryotic cells is coated with proteins and forms a highly compact and dynamic chromatin structure. Interactions between DNA and proteins are important for numerous cellular processes, such as cell division, transcription, and replication. These interactions are mostly transient and dynamic, guaranteeing that these remarkable complex reactions occur in a time- and space-regulated manner. However, proteins can be accidently covalently linked with DNA molecules, which can block not only interactions between other proteins and DNA but also DNA transactions that must slide-through DNA molecules. We call this covalent, irreversible binding of protein to DNA a DNA–protein cross-link (DPC), which is considered a type of DNA damage.

The first report of DPCs in living cells was in 1962, when researchers found that the extractability of bacterial DNA from these cells after ultraviolet irradiation decreased in a dose-dependent manner [1]. It was realized later that DPCs can be induced by a lot exogenous and endogenous agents, such as ionizing radiation, ultraviolet light, metals and metalloids, aldehyde, and chemotherapeutic drugs [2–5]. These agents induce DPCs via distinct chemical mechanisms, resulting in various types of DPCs [2]. These covalently DNA-bound proteins pose a physical challenge to all types of DNA transactions and are therefore harmful to cells. Thus, knowing how DPCs form in different situations, the consequences of DPCs, how cells deal with DPCs, and how we can use the underlying knowledge for cancer therapy is important.

Depending on the properties of DPCs, which are diverse, cells employ different repair pathways to deal with them. Investigators have shown that nucleotide excision repair (NER) and homologous recombination (HR) target the damaged DNA and remove DPCs with different size limits for proteins [6–11]. Direct reversal of specific DPCs by hydrolysis, chelation, and targeted enzymes like tyrosyl-DNA phosphodiesterase 1 (TDP1) and TDP2 were also reported [12]. However, repair mechanisms that target covalently bound proteins were not clear until the discovery of the proteases Wss1 in yeast and SprT-like domain at the N-terminus (SPRTN) in humans [13–18]. Wss1 and SPRTN, which is also known as C1orf124, SPARTAN, or DVC1 (DNA damage-targeting VCP p97 adaptor C1orf124), can directly degrade proteins that are covalently bound to DNA and allow other repair factors to access the damage sites. Studies have also implicated involvement of proteasomes in the degradation of the covalently bound proteins [19, 20], but the detailed mechanism of how it functions remains unclear. Herein we summarize the progress in the DPC repair field and describe how cells may employ these different repair pathways for efficient repair of DPCs.

## Types of DPCs

**Fig. 1:**
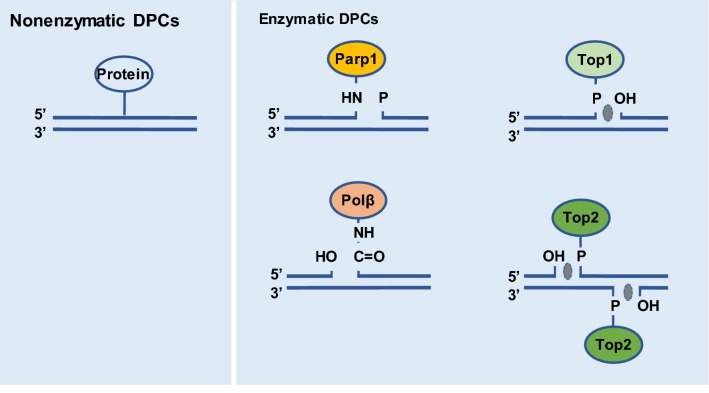
DPCs can be categorized as nonenzymatic or enzymatic based on the properties of the cross-linked proteins. Any proteins located in the vicinity of DNA can result in nonspecific DPCs triggered by various agents, including reactive compounds like aldehydes, metal ions, and several types of radiation. These are defined as nonenzymatic DPCs. Also, many DNA-related enzymatic reactions produce intermediates in which transient covalent linking between DNA and the enzyme occurs. Enzymes, such as DNA TOPs, DNA polymerases, and DNA methyltransferases, can be trapped and therefore form stable DPCs under certain circumstances. These are defined as enzymatic DPCs

Unlike other types of DNA lesions, DPCs can be produced by any nuclear proteins that are located in the vicinity of DNA and therefore could be cross-linked with DNA [21, 22]. Based on the properties of cross-linked proteins, DPCs can be classified as enzymatic or nonenzymatic (Fig. 1) [23, 24].

### Enzymatic DPCs

Many DNA-related enzymatic reactions produce intermediates in which transient covalent linking of DNA with an enzyme occurs. Typically, the enzymes involved in such reactions are DNA topoisomerases (TOPs), DNA polymerases, DNA methyltransferases, DNA glycosylases, or apurinic or apyrimidinic lyases (Fig. 1) [25–27]. Generally, these intermediates are not stable, and the covalent linking can be reversed very quickly. However, under certain circumstances, such transient intermediates can be trapped, thereby forming stable DPCs. The most well-known enzymatic DPCs are the covalent links between DNA and TOPs. Specifically, TOP1 relieves the torsional stress of DNA supercoiling by cleaving on a single strand of DNA. The 3′ end of the resulting single-strand break is covalently bound to TOP1, whereas the 5′-OH end is free and can rotate around the intact DNA strand to release the torsional stress. Afterward, TOP1 catalyzes annealing of the single-strand break and is then released from DNA. However, TOP1-dependent annealing of single-strand breaks can be easily inhibited because successful ligation of the breaks can only be achieved if the two DNA ends or strands are properly aligned. This means that any distortion of the DNA structure that disturbs the alignment of DNA strands will lead to permanent trapping of TOP1 and therefore formation of a stable DPC at the site of the single-strand break. Typically, such distortion of DNA strands can be caused by nearby DNA lesions like abasic sites. Alternatively, small molecules like camptothecin and its derivatives used in chemotherapy may prevent ligation of these strands [28]. Similarly, TOP2 can be trapped in DNA and contribute to the formation of DPCs [29]. Because TOP2 induces double-strand breaks (DSBs), the TOP2-associated DPCs are generally located at the terminal ends of DSBs. Therefore, enzymatic DPCs are normally accompanied by DNA lesions, such as single-strand DNA breaks for TOP1 and DSBs for TOP2.

### Nonenzymatic DPCs

Besides particular enzymes surrounding DNA strands, other proteins located in the vicinity of DNA can result in nonspecific DPCs under certain circumstance (Fig. 1). Cross-linking of proteins with DNA to form these nonenzymatic DPCs can be triggered by various agents, including reactive compounds like aldehydes, metal ions, and several types of radiation [3, 30–33]. Regarding aldehydes, formaldehyde (FA) is generated from histone demethylation [30], and acetaldehyde is a metabolic product of ethanol oxidation [34]. FA produces DPCs by forming methylene bridges between DNA bases and nucleophilic amino acid residues [30, 35, 36]. The mechanisms underlying ionizing radiation-induced DPC formation are unclear, but researchers have suggested that this kind of DPC formation has important clinical potential [37–39]. As far as we know, ionizing radiation leads to radiolysis of water molecules, which results in high levels of free radicals and reactive oxygen species in a locally restricted environment. These highly reactive species trigger multiple types of DNA lesions, including DPCs. Nonenzymatic DPCs normally involve proteins attached to undisrupted DNA strands and are therefore very different from enzymatic DPCs, especially TOP-associated DPCs.

## Mechanisms of DPC repair

**Fig. 2:**
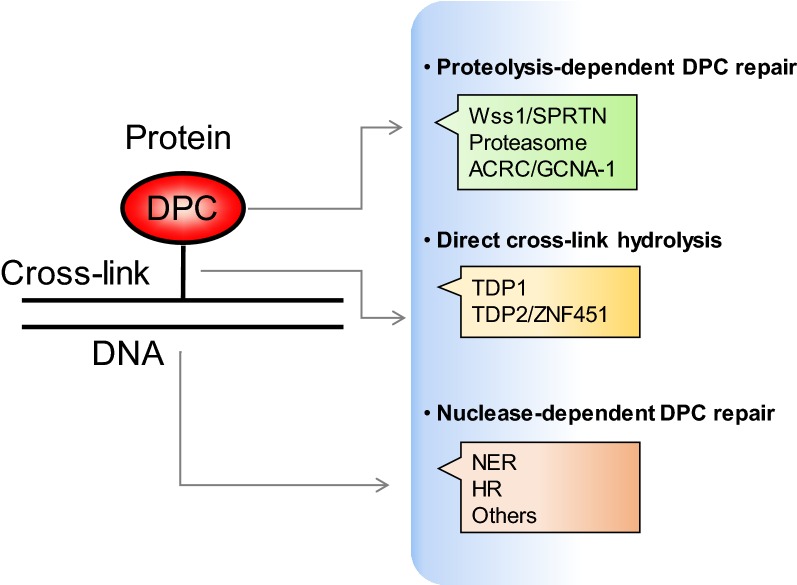
DPCs are composed of DNA, protein, and their cross-linked bonds, which can be targeted by different repair pathways. NER and HR are nuclease-dependent pathways that can directly cleave DNA molecules. The chemical bond between TOP1/TOP2 and DNA can be directly hydrolyzed by TDP1 and TDP2/ZNF451. Also, proteasomes, SPRTN/Wss1, and ACRC/GCNA-1 are related to proteolysis-dependent removal of covalently bound proteins

As stated above, DPCs are composed of DNA, protein, and cross-linked bonds of them [40] and can arise via different mechanisms, which results in diversity of any of the three DPC components. Cells likely cannot detect DPCs using highly specific sensors. Several repair pathways are reported to be involved in the repair of DPCs [12, 23, 24, 33, 40, 41]. Below we summarize these repair pathways, placing them in three categories based on the DPC components they target (Fig. 2).

### Nuclease-dependent repair mechanisms targeting DNA molecules: NER, HR, and others

The first insight into the involvement of NER and HR in DPC repair came in early genetic studies of *Escherichia coli*. By characterizing the survival and mutagenic effects of DPC-inducing agents like FA and 5-aza-2′-deoxycytidine, researchers found that *uvrA* and *recA* mutants, which are defective in NER and HR, respectively, were sensitive to FA-based treatment [42, 43]. However, the *recA* but not the *uvrA* mutants were sensitive to treatment with 5-aza-2′-deoxycytidine [44, 45]. Later, several lines of biochemical and genetic evidence further demonstrated that the NER and HR pathways cooperate closely but commit differentially to DPC repair [9, 10]. NER repairs DPCs with cross-linked proteins smaller than 12–14 kDa, whereas HR mainly repairs oversized DPCs. The limitation of NER in repairing oversized proteins is determined by the loading efficiency of UvrB, which influences the incision efficiency of DNA by UvrABC complex during NER [10]. Similarly, genetic studies with yeast demonstrated the involvement of the NER and HR pathways in the repair of FA-induced DPCs, with NER having a dominant role in repair following treatment with acute high doses of FA and HR aiding repair following treatment with chronic low doses of FA [6]. NER also seems to eliminate particular types of DPCs in mammalian cells [11, 46]. However, because the size of the cross-linked protein in NER based DPC removal is limited to 8–10 kDa, employment of NER alone in repairing DPCs in vivo is limited [47]; preprocessing of the cross-linked protein by a proteasome or protease may be required.

The involvement of HR in DPC repair seems to be conserved in mammalian cells [48, 49]. Mammalian cells treated with FA accumulate DSBs and RAD51 foci and also have increased rates of sister chromatin exchange events, all of which indicate an activated HR pathway [50]. Unlike with the direct digestion of DNA around DPCs by NER, evidence of the function of HR regarding intact DPCs is lacking. The involvement of HR in repair of intact DPCs likely depends on the formation of DSBs near DPCs. One example for this is the MRE11, RAD50, and NBS1 (MRN) complex [51], which is an important nuclease complex in the initiation of resection of the HR pathway. Use of the MRN complex in resolving DNA ends correlates with its evolutionally conserved role in DPC repair [7, 52–54]. In particular, repair of antitumor agent-induced TOP-DNA cross-links in T4 bacteriophages was dependent on the MR complex (i.e., gp46/47) [52, 53]. Also, the *E. coli* SbcCD (MR) complex was able to nucleolytically process protein-bound DNA ends [54]. Similarly, in yeast, Mre11-deficient strains were highly sensitive to treatment with TOP inhibitors [55]. In addition, DSBs with proteins covalently bound to the 5′ termini ends generated by Spo11 during meiotic recombination were endonucleolytically cleaved by the Mre11/Rad50/Xrs2 (homologs of MRN) complex, resulting in the release of Spo11 attached to an oligonucleotide [7, 56–58]. As a note, the yeast meiotic specific protein Spo11 shares sequence homology with archaeal topoisomerase VI and reacts just like topoisomerase to generate Spo11-DNA intermediate. Similarly, biochemical analysis of *Xenopus* egg extracts demonstrated the cooperation of the MRN complex, CtIP, and BRCA1 in removal of Top2-DNA covalent adducts and subsequent resection of DSB ends [59]. Consistent with these observations, the MRN complex also facilitates removal of TOP2-DNA covalent adducts from mammalian cells [60, 61]. However, deletion of MRE11 in mammalian cells by small interfering RNA did not increase the total number of DPCs formed in vivo under unperturbed conditions [16], demonstrating that multiple pathways may be involved in the processing and repair of these DPCs.

The nuclease-dependent DPC repair mechanisms targeting DNA molecules are restricted by the accessibility of nucleases to substrates. Large proteins (> 8–10 kDa) can block loading of the NER repair machinery and reduce the incision efficiency of NER nucleases. Preprocessing pathways that can reduce the protein size or relax the structure of bound proteins may be needed before the NER pathway can access and repair these DPCs. Additionally, DPCs without any DNA ends cannot be recognized by an MRN-directed HR pathway. Prenucleolytic cleavage of DNA by other pathways, such as NER, may produce a substrate that can be subsequently repaired by the HR pathway. Therefore, evaluating the participation of NER and/or HR in DPC repair is critical, as their involvement in this repair may vary according to the type of DPC.

### Hydrolysis of the chemical bond between proteins and DNA by TDP1 and TDP2/ZNF451

As mentioned above, the chemical bonds between proteins and DNA in DPCs are quite diverse, which makes involvement of a specific enzyme in reversing each type of covalent bonds impossible. However, some types of enzymatic DPCs occur frequently, and cells have evolved specific enzymes to induce direct hydrolysis of these chemical bonds. For example, TDP1 and TDP2 are two enzymes that can specifically reverse covalent bonds of DNA with TOP1 and TOP2, respectively [12].

Researchers first identified TDP1 in yeast based on its activity in hydrolyzing phosphotyrosyl bonds at the 3′ ends of DNA [62, 63]. Also, studies demonstrated that TDP1 repairs covalent TOP1-DPCs in vivo [63, 64]. TDP1 is conserved in eukaryotic cells, and deficiency of TDP1 confers sensitivity to TOP1 inhibitors in cells and in organisms ranging from yeast to humans [64–71]. TDP1 not only can hydrolyze 3′-tyrosine but also is active against a wide range of other 3′ DNA end-blocking adducts, such as those produced by oxidative DNA damage [12]. TDP1 functions as a monomer and processes its substrates via formation of transient covalent intermediates [72, 73]. After hydrolysis by TDP1, the DNA has a 3′-phosphate end, which must be further processed by polynucleotide kinase phosphatase to generate a 3′-hydroxyl end that can be extended by polymerases. Mutations in the TDP1 catalytic domain result in accumulation of TDP1-DNA intermediates and lead to the rare autosomal recessive neurodegenerative disease spinocerebellar ataxia with axonal neuropathy [69, 74].

Researchers discovered the function of TDP2 in repairing DPCs in a genetic screen designed to identify suppressors of camptothecin sensitivity in *tdp1*- and *rad1*-deficient yeast cells with expression of human cDNAs [75]. TDP2 exhibited prominent activity toward 5′-tyrosyl DNA ends [75, 76], and cells deficient in TDP2 were hypersensitive to treatment with TOP2 inhibitors [75–78]. Although investigators have broadly identified homologs of TDP2 in eukaryotic cells, yeast homologs have yet to be discovered. Unlike for TDP1, two divalent metals are required for TDP2′s catalytic activity, and TDP2 does not form covalent-linked intermediates [75, 79, 80]. TDP2 generates 5′-phosphate DNA ends, which can be directly processed by ligases. Homozygous mutations of the TDP2 gene were associated with spinocerebellar ataxia autosomal recessive 23, a disease characterized by intellectual disability, seizures, and ataxia [77].

Similar to the nuclease-dependent DPC repair pathways, TDP1 and TDP2 are restricted by the accessibility to substrates, which are easily buried by covalently bound proteins. Both TDP1 and TDP2 were unable to remove full-length TOP1 or TOP2 and needed prehydrolysis of these proteins by a proteasome [77, 81–84]. However, a recent study demonstrated that the small ubiquitin-related modifier (SUMO) ligase ZATT (ZNF451) can mediate direct resolution of the TOP2-DNA covalent complex (TOP2-cc) by TDP2 [85]. Researchers showed that ZNF451 can directly bind to and SUMOylate TOP2-cc, which enhances the hydrolase activity of TDP2 and promotes its efficient recruitment to damage sites [85]. Further studies are needed to identify any other mechanisms of promoting the direct hydrolytic activity of TDP1 and TDP2 toward TOP1-cc and TOP2-cc, respectively.

### Proteolysis-dependent repair mechanisms targeting cross-linked proteins: proteasomes, SPRTN/Wss1, and acidic repeat-containing protein/germ cell nuclear antigen-1

Proteolysis of covalently bound proteins during DPC repair has been observed for quite some time [19, 81, 84, 86, 87] and originally attributed to the function of proteasomes. The 26S proteasome is the principle proteolytic machine for regulated protein degradation in eukaryotic cells [88, 89]. Normally, proteins are marked by polyubiquitin chains before they are recognized and degraded by proteasomes [88, 89]. Indeed, researchers observed ubiquitination of TOP1 after treating cells with TOP1 inhibitors [81, 87, 90]. Also, blockage of proteasome activity by inhibitors like MG132 and lactacystin hindered the proteolysis of TOP1-cc [81, 87, 90]. Furthermore, degradation of TOP1 was blocked when the E1 ubiquitin-activating enzyme was inactivated in ts85 cell lines [81, 87, 90]. Investigators also observed proteasome-dependent degradation for of TOP2-cc [84] and FA-induced DPCs [19]. However, deficiency of cytosolic ATP-dependent proteases in bacteria, which are the counterparts of eukaryotic proteasomes, did not affect cell survival following treatment with FA or 5-aza-2′-deoxycytidine [10]. A study using *Xenopus* egg extract demonstrated that inhibition of proteasome activity had no obvious effect on DPC repair in vitro, but that adding ubiquitin-vinyl sulfone, a deubiquitylation enzyme inhibitor, blocked the degradation of proteins in DPCs [91]. Moreover, adding free ubiquitin back to the reaction restored the destruction of proteins in DPCs [91]. Therefore, the authors concluded that the presence of free ubiquitin but not the activity of deubiquitylation enzymes or proteasomes is required for the repair of DPCs. These contradictory conclusions may be due to the use of proteasome inhibitors for the experiments, which not only inhibit proteasome activity but also deplete the free ubiquitin pool that may affect other ubiquitin-dependent functions. More recently, a study using an in vitro DPC repair system identified the accumulation of proteasome proteins on replicating DPC plasmids and found that proteasome-mediated degradation of polyubiquitinated DPCs requires the action of the E3 ligase TRAIP [92]. Further studies are needed to define the exact roles of proteasomes in DPC repair in vivo.

In recent years, investigators identified a more specific proteolytic pathway with the finding of Wss1 in yeast cells and SPRTN in mammalian cells. Wss1, a weak suppressor of *smt3*-*331*, is a metalloprotease that was first linked with the SUMO pathway in yeast [93, 94]. The discovery of Wss1 functions in DPC repair came in a synthetic interaction screening of a tdp1-knockout yeast strain [13]. Researchers found that co-deletion of wss1 and tdp1 led to extremely slow growth of yeast cells and hypersensitivity to camptothecin treatment, which could be relieved by deletion of Top1 [13]. Further in vitro biochemical studies showed that Wss1 can cleave the DNA-binding protein Top1, histone H1, high mobility group protein 1, and itself in a DNA-dependent manner. Cells lacking wss1 were hypersensitive to FA-based treatment. Additionally, interaction studies demonstrated that Wss1 works with Cdc48 in processing genotoxic SUMO conjugates [13, 95]. Recent report also indicated the involvement of Wss1 in DNA replication stress response [96]. They found that deletion of wss1 in yeast sensitized cells to hydroxyurea-based treatment and that further deletion of another protease, ddi1, made the cells even more sensitive to this treatment, suggesting a strong genetic interaction between wss1 and ddi1 [96, 97]. However, whether the proteolytic activity of Wss1 is required for its involvement in replication stress response has yet to be addressed.

In a bioinformatic analysis based on sequence similarity and domain organization, researchers speculated that SPRTN is a functional homolog of Wss1 [24]. Both SPRTN and Wss1 contain a protease domain with a conserved HEXXH active site and harbor the motif responsible for interaction of the protein with the segregase Cdc48 (p97 in higher eukaryotes). Moreover, both Wss1 and SPRTN contain modification-directed binding domains, a SUMO-interacting motif, or the ubiquitin interaction domain UBZ, respectively. SPRTN also harbors a proliferating cell nuclear antigen (PCNA)-interacting motif (PIP box), which directs its binding to PCNA. Indeed, more recent studies revealed a similar function of SPRTN in proteolysis of proteins on DPCs [14–18].

However, before discovery of its function in DPC repair, SPRTN was first characterized as a PCNA interacting protein involved in translesion synthesis [98–104]. SPRTN can be recruited to DNA damage sites via a PIP box and UBZ domain [98–104]. Conflicting results showed the dependence of damage-induced SPRTN localization on RAD18 and PCNA ubiquitin [100–102, 104] and the independence of this localization on them [98, 99]. Knockdown of SPRTN sensitized cells to treatment with ultraviolet radiation and increased mutagenesis during replication of ultraviolet radiation-damaged DNA [98–104]. SPRTN also interacts with VCP/p97 via the SHP domain [98–104]. Whether SPRTN promotes the recruitment of Polη to damage sites (TLS polymerase) [101, 102] or its release from damage sites [98, 99] is under debate.

Notably, biallelic germline mutations in SPRTN have caused Ruijs–Aalfs syndrome, a human autosomal recessive disorder characterized by genomic instability and early-onset hepatocellular carcinoma [105]. Also, SPRTN insufficiency in mice recapitulated some of the characteristics of human patients with Ruijs–Aalfs syndrome, such as chromosomal instability, premature aging, and early-onset age-related phenotypes [17, 106]. In vivo studies revealed that SPRTN-deficient cells are hypersensitive to treatment with DPC-inducing agents, are defective in removing DPCs, and accumulate nonspecific and TOP-involved DPCs in vivo due to defective protease activity [14–18]. In vitro biochemical assays further proved that SPRTN is a protease that can degrade histones, TOP, and itself in a DNA-dependent manner [14–18]. Studies also suggested that SPRTN travels with the replication fork and removes DPCs depending on the presence of DNA replication [16, 91]. Furthermore, the protease activity of SPRTN is tightly regulated with a switch that depends on its DNA binding, ubiquitination, and autocleavage [14–18]. Both single- and double-stranded DNA can activate the protease activity of SPRTN, with single-stranded DNA being more effective [14–16, 107]. SPRTN can be monoubiquitinated, but only unmodified SPRTN binds to chromatin [15]. Therefore, investigators proposed that DPCs somehow cause SPRTN deubiquitination, which promotes the binding of SPRTN to DNA and its activation [15]. Researchers have also observed autocleavage of SPRTN, which they proposed to be a mechanism of its tight regulation and prevention of unnecessary degradation of proteins other than DPCs on chromatin [14–16, 107]. Whether some or all of these mechanisms are involved in the regulation of SPRTN function remains to be determined.

Structure analysis showed that the catalytic centers of Wss1 and SPRTN are highly solvent-exposed and lack a substrate-binding cleft, which can explain the lack of specificity of their activity [15, 107, 108]. A recent study reported that SPRTN can degrade nonubiquitylated DPCs [92]. Thus, how SPRTN acts with VCP/p97 segregase and/or proteasomes must be investigated further.

A more recent study proposed that acidic repeat-containing (ACRC) protein is an SPRTN-related protease [41]. It contains a conserved catalytic domain just as those in Wss1 and SPRTN and is in proximity to SPRTN based on phylogenetic analysis results [41]. In a comprehensive proteomic profiling study aimed at characterizing SUMOylation response to DPC induction in human cells, researchers showed that ACRC protein interacted with a polySUMO chain and could be recruited to FA-induced foci, which was dependent on SUMOylation [109]. In addition, in *Caenorhabditis elegans*, the ACRC protein ortholog germ cell nuclear antigen (GCNA)-1 promoted survival after DPC induction [109]. Determining whether ACRC protein and GCNA-1 function as proteases in proteolysis of DPCs in vivo and how they may interplay with Wss1 and SPRTN requires further experimentation.

Even after proteolysis by a proteasome or Wss1/SPRTN, DPCs are not fully removed from DNA strands [91]. Small peptides are left covalently bound to the DNA, which can be further processed by NER, HR, or TDP1/TDP2. In addition, bypass of peptide-DNA conjugates may rely on the translesion synthesis pathway [91].

## Conclusions

The finding of specific proteases such as Wss1 and SPRTN in direct proteolysis covalently bound proteins inspires the current working hypothesis that a specific DPC repair pathway exists in vivo. Insightful mechanistic studies of Wss1 and SPRTN may help uncover their “co-workers” in DPC repair and provide a comprehensive understanding of this specific DNA repair pathway. Questions remain about how cells choose different repair pathways, including NER, HR, TDP1/TDP2, proteasomes, and Wss1/SPRTN, for DPC repair and how these pathways may interplay with each other. Given the critical roles of DPC repair in the physiological setting as well as following treatment with many antitumor modalities, DPC repair is likely a meaningful target for cancer treatment, especially in combination with inhibition of other repair and/or checkpoint pathways.

## Data Availability

Not applicable.

## Abbreviations

ACRC: acidic repeat-containing
DPC: DNA–protein cross-link
DSB: double-strand break
FA: formaldehyde
GCNA: germ cell nuclear antigen
HR: homologous recombination
MRN: MRE11, RAD50 and NBS1
NER: nucleotide excision repair
SUMO: small ubiquitin-related modifier
SPRTN: SprT-like domain at the N terminus
TDP: tyrosyl-DNA phosphodiesterase
TOP: topoisomerase
TOP2-cc: TOP2-DNA covalent complex
